# Global Oncology Medical Diplomacy Working Group Inaugural Meeting: Defining Worldwide Barriers to Germline Genomics in Cancer Prevention and Management

**DOI:** 10.5334/aogh.3967

**Published:** 2023-02-21

**Authors:** Ghassan K. Abou-Alfa, Larry Norton, The Global Oncology Medical Diplomacy Working Group

**Affiliations:** 1 Memorial Sloan Kettering Cancer Center, US

**Keywords:** germline, cancer, global oncology, global health, medical diplomacy

## Abstract

We convened an international working group to examine the issues that challenge equity and inclusion in genetic medicine. Specifically, 72 internationally known experts in oncology and cancer genetics from 34 countries (the Global Oncology Medical Diplomacy Working Group), gathered virtually on January 4–5, 2022, for the “Humanity Cancer Germline Convergence and Divergence Cancer Predispositions” conference hosted by Memorial Sloan Kettering Cancer Center, in collaboration with the United Arab Emirates Ministry of Health and the Al Jalila Foundation. The goal of the conference was to broaden transnational understanding of the current state of genetics in preventive and therapeutic cancer medicine, and to define barriers to increased uptake of germline genomics to decrease the international burden of cancer. Here, we highlight the overarching barriers that were defined through this effort. These global barriers to incorporating germline genomics into optimal cancer care can inform ongoing research, collaboration, and advocacy for equitable, cost-effective genomic medicine for populations worldwide.

As it is increasingly recognized that preventive approaches could have a substantial impact on cancer mortality worldwide, hereditary and environmental risk factors provide an important target for intervention. It is estimated that over half of cancer deaths could be prevented by addressing smoking and known environmental, dietary, infectious, and other causes of cancer [1]. Hereditary factors account for approximately 16% of cancers [2,3], leading to approximately 91,000 deaths in the US each year. Worldwide, approximately 18.1 million people are diagnosed with cancer, resulting in 9.6 million deaths annually [4], 1.5 million of which may be associated with hereditary factors. Over the last two decades, the identification of highly penetrant cancer susceptibility genes, as well as lower risk but more common genomic variants, have provided new tools for genetic counseling, prevention, as well as targeted treatment of hereditary cancers [5,6,7]. In countries with great healthcare access, there are clear examples of successful early detection, prevention, and risk reduction programs, and therapeutic implications have been reported [8,9,10,11]. Thus further highlight the recognition of inequities in cancer genetic testing uptake in high income countries has resulted in strategies to improve access and attempt to overcome disparities in the provision of genetic care [12,13,14]. Despite such important implications of genomic factors in oncology, persistent global barriers limit equitable access, uptake, and efficacy of cancer genetics as a tool for public health. Overcoming barriers to incorporating germline information in oncologic care can improve clinical decision-making and preventative as well as therapeutic applications. By putting patients first, we hope that this effort will foster interpersonal and interprofessional relationships that will emphasize the connectedness of people even in areas of conflict, and thus nurture attitudes toward peace.

To begin to define and address barriers to equitable diffusion and utilization of genomics in cancer prevention and management practices worldwide, we convened an international working group to examine the complex, potentially sensitive issues that challenge equity and inclusion in genetic medicine. The meeting was organized so, as to include worldwide representation with careful attention to including colleagues in areas of conflict. We choose medical oncologists and geneticists who were active in clinical and/or research activities as assessed by publications and participation in international meetings, organizations, and professional societies. The final group included 72 internationally known experts in oncology and cancer genetics from 34 countries (the Global Oncology Medical Diplomacy Working Group; Figure 1 and Table 1), gathered virtually on January 4–5, 2022, for the “Humanity Cancer Germline Convergence and Divergence Cancer Predispositions” conference hosted by Memorial Sloan Kettering Cancer Center, in collaboration with the United Arab Emirates Ministry of Health and the Al Jalila Foundation (agenda in Supplemental Table 1). This first-of-its-kind event united global attendees under a common goal: to broaden transnational understanding of the current state of genetics in preventive and therapeutic cancer medicine, and to define barriers to increased uptake of germline genomics to decrease the international burden of cancer. The conference encompassed parallel break-out sessions dedicated to open discussion among representatives of five geographic regions: Africa, the Americas, Asia, Eurasia, and the Middle East. This was followed by a final whole-group open discussion of themes and next steps. Based on the rich dialogue that ensued, we highlight the overarching barriers that were defined.

**Table 1:** Name, affiliation, and country (alphabetical order) of expert members of the Global Oncology Medical Diplomacy Working Group.


NAME	AFFILIATION	COUNTRY

Mohammed Oukkal	Beni-Messous University	Algeria

Angela Solano	Centro de Educacion Medica e Investigaciones Clinicas (CEMIC) University of Buenos Aires/CONICET	Argentina

AFM Kamal Uddin	National Institute of ENT	Bangladesh

Sergei Krasny	N.N. Alexandrov National Cancer Centre of Belarus	Belarus

Maria Isabel Achatz	Hospital Sírio-Libanês	Brazil

Bruno Nervi	Pontificia Universidad Católica de Chile	Chile

Tony Mok	The Chinese University of Hong Kong	China

Qing Zhou	Guangdong Lung Cancer Institute, Guangdong Provincial People’s Hospital, Guangdong Academy of Medical Sciences, Guangzhou, China	China

Nermine Kamal	Cairo University	Egypt

Mohsen Mokhtar	Cairo University	Egypt

Endale Hadgu Gebregzabher	St. Paul’s Hospital Millennium Medical College	Ethiopia

Fabrice Andre	Gustave Roussy Cancer Center	France

Lama Sharara	A.R.CA.D Foundation	France

Rajiv Sarin	Tata Memorial Hospital	India

Bhawna Sirohi	Apollo Proton Cancer Centre, Chennai	India

Talia Golan	Chaim Sheba Medical Center	Israel

Ephrat Levy-Lahad	Shaare Zedek Medical Center, Faculty of Medicine, The Hebrew University of Jerusalem	Israel

Takeshi Kuwata	National Cancer Center Hospital East	Japan

Hikmat Abdel-Razeq	King Hussein Cancer Center	Jordan

Sana Al-Sukhun	Al Hyatt Oncology Practice	Jordan

Dilyara Kaidarova	Kazakh Institute of Oncology and Radiology	Kazakhstan

Marwan Ghosn	Saint Joseph University in Beirut	Lebanon

Naji El Saghir	American University of Beirut	Lebanon

Soo-Hwang Teo	Cancer Research Malaysia	Malaysia

Rosa Maria Alvarez Gomez	Instituto Nacional de Cancerologia	Mexico

Erika Ruiz -Garcia	Instituto Nacional de Cancerologia	Mexico

Sheila Mabote	Fernandes Figueira National Institute of Women	Mozambique

Abeer Alsayegh	Sultan Qaboos University	Oman

Samir Fasih	Shaukat Khanum Memorial Cancer Hospital and Research Centre	Pakistan

Muhammad Usman Rashid	Shaukat Khanum Memorial Cancer Hospital and Research Centre	Pakistan

Neelam Siddiqui	Shaukat Khanum Memorial Cancer Hospital	Pakistan

Basim Ayesh	Al Aqsa university-Gaza	Palestine

Moien Kanaan	Bethlehem University	Palestine

Rami Musallam	Islamic University of Gaza	Palestine

Reem Al Sulaiman	Hamad Medical Corporation	Qatar

Salha Bujassoum	Hamad Medical Corporation	Qatar

Vsevolod Matveev	N.N.Blokhin National Cancer Research Center	Russia

Mohammed Algarni	King Abdulaziz Medical City. Ministry of National Guard Health Affairs (MNGHA)	Saudi Arabia

Omalkhair Alkhair	Alhabib Hospital. Adjunct Associate Prof Alfaisal University	Saudi Arabia

Sultan Sedairy	King Faisal Specialist Hospital & Research Centre	Saudi Arabia

Rebecca Dent	National Cancer Center Singapore	Singapore

Joanne Ngeow	Nanyang Technological University Singapore	Singapore

Maritha J Kotze	Stellenbosch University & National Health Laboratory Service	South Africa

Jeong Eun Kim	Asan Medical Center	South Korea

Maha Manachi	Albairouni University Hospital	Syria

Maher Saifo	Damascus University	Syria

Gokmen Aktas	Medical Park Gaziantep Hospital Oncology Center	Turkey

Mehmet Ali Yavuz	Nizip Community Hospital	Turkey

Amin Alamiri	UAE Ministry of Health and Prevention	United Arab Emirates

Abdulkareem Al Olama	AlJalia Foundation	United Arab Emirates

Shaheenah Dawood	Dubai Health Care City	United Arab Emirates

David Cameron	University of Edinburgh	United Kingdom

Ghassan Abou-Alfa	Memorial Sloan Kettering Cancer Center	United States of America

Judy Garber	Harvard Dana Farber	United States of America

Mrinal Gounder	Memorial Sloan Kettering Cancer Center	United States of America

Rachel Grisham	Memorial Sloan Kettering Cancer Center	United States of America

David Kelsen	Memorial Sloan Kettering Cancer Center	United States of America

Bob T. Li	Memorial Sloan Kettering Cancer Center	United States of America

Ying Liu	Memorial Sloan Kettering Cancer Center	United States of America

Sophia Michaelson	American Eurasian Cancer Alliance (AECA)	United States of America

Larry Norton	Memorial Sloan Kettering Cancer Center	United States of America

Kenneth Offit	Memorial Sloan Kettering Cancer Center	United States of America

Funmi Olopade	University of Chicago	United States of America

Eileen O’Reilly	Memorial Sloan Kettering Cancer Center	United States of America

Philip Philip	Wayne State University School of Medicine	United States of America

Lewis Roberts	Mayo Clinic	United States of America

Mark Robson	Memorial Sloan Kettering Cancer Center	United States of America

Andrew Seidman	Memorial Sloan Kettering Cancer Center	United States of America

Rania Sheikh	Memorial Sloan Kettering Cancer Center	United States of America

Zsofia Stadler	Memorial Sloan Kettering Cancer Center	United States of America

Tanya Trippett	Memorial Sloan Kettering Cancer Center	United States of America

Dilshod Egamberdiev	National Cancer Center of Uzbekistan	Uzbekistan


**Figure 1:**
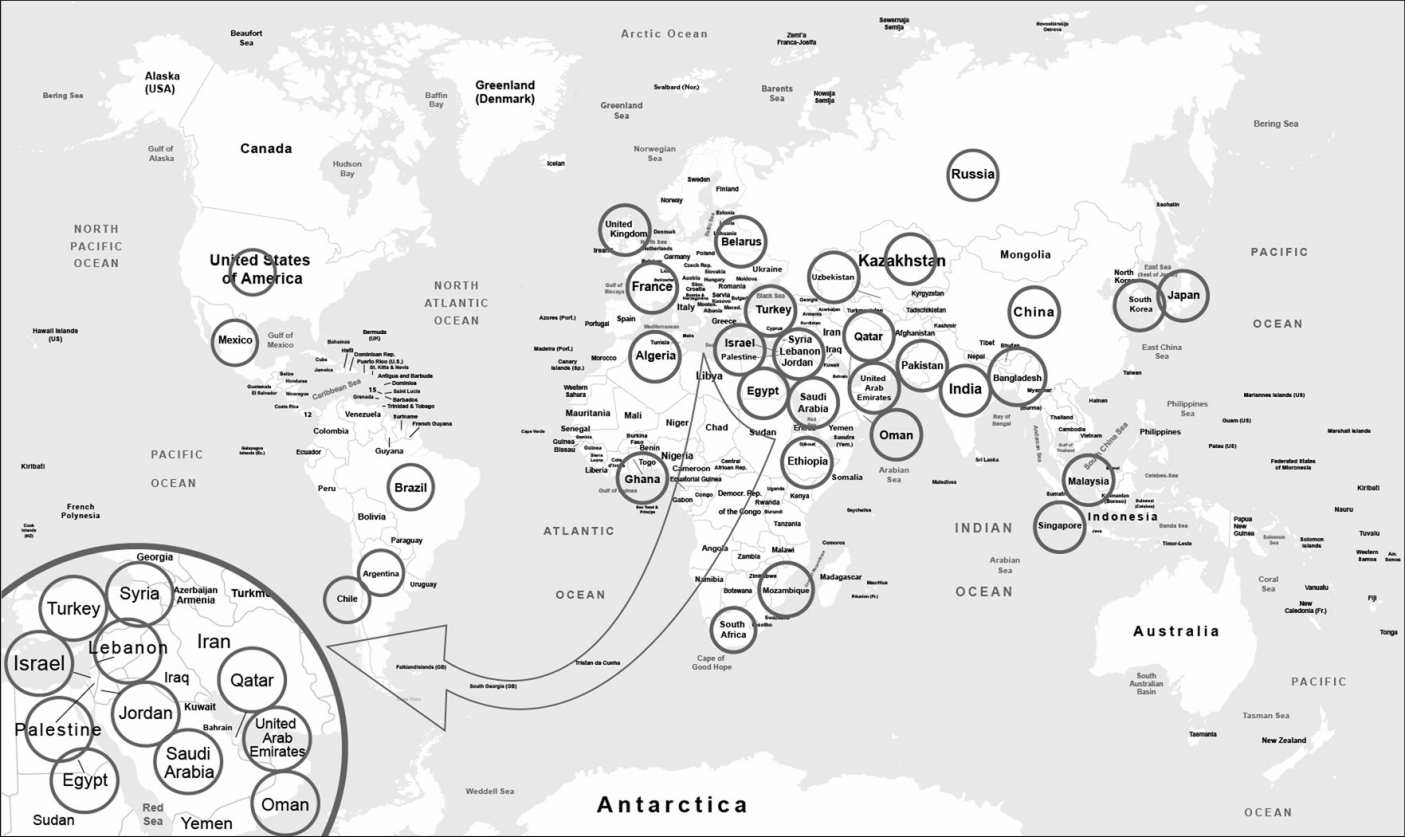
Countries represented at the 2022 Global Oncology Medical Diplomacy Working Group conference.

Several broad themes emerged, including the need for greater knowledge of population-specific differences in inherited genetic variants; widely variable access and uptake of genetic testing in clinics and in populations; disparate availability of genomic testing technologies; incomplete use of germline genetic results to inform cancer care; and inequities in access to high-cost therapeutics targeting genomic pathways. These factors were deemed as most salient in limiting the impact of genomic strategies that can inform oncology practice, as well as emerging approaches to identify cancer predisposing variants in extended families. Such “cascade” testing has been proposed [15] as a cost-effective alternative to population testing [16].

Overall, the group emphasized the need for greater knowledge of population-specific differences in inherited genetic variants. While a readily accessible database of variants (i.e., CLINVAR, https://www.ncbi.nlm.nih.gov/clinvar/) describes pathogenic variants, including founder mutations, in cancer predisposition genes, with some exceptions (e.g., *TP53*) [17] the description is more limited in the global south (e.g., Middle East, large parts of Asia, and the Pacific Rim). Despite global efforts to catalog variants in some genes (e.g., the *BRCA1/2* Global Alliance) [18], there was a perceived need for greater efforts to measure overall burden of genetic variants across multiple genomic pathways, as well as across continents.

Conference members also observed that current genomic sequence data for cancer predisposition genes are largely based on studies of populations from North America and European descent, who have the highest access to testing. Thus, a substantial proportion of the hereditary burden remains to be discovered worldwide, and the prevalence/penetrance of known mutations in specific populations is poorly characterized. Knowledge of founder mutation or population-specific pathogenic variants varies widely, from fully deployed consanguinity screening in some countries to individual efforts in other countries in the same region. Recognition of potential convergence and divergence of populations underscored the need for a deeper understanding of fundamental genotype-phenotype correlations in diverse populations, and the need to identify population-specific genomic and environmental modifiers.

Variable access and uptake of genetic testing in clinics and in populations, and unequal availability of genomic testing technologies were common between regions. It was noted that there is substantial variability in technologies employed worldwide. While most laboratories use massively parallel sequencing (MPS) platforms to identify variants, these laboratories differ with regard to methods and, importantly, deposition of variants into public databases [19]. Some participants voiced concern over cutbacks in governmental support of centralized laboratories and the need for greater coordination and sharing of expertise among testing laboratories.

Although social, cultural, and societal barriers to genetic testing are well-recognized, frank discussion in this forum defined these aspects to be considerable, and in some cases profoundly challenging. Concerns of discrimination, stigma, and use of data for political/economic reasons were potential factors contributing to patients declining testing in all regions [6,7]. The lack of protection against genetic discrimination – that is, patients’ fear of discrimination by insurance companies, employers, and society stemming from results of genetic testing – was highlighted in several discussions [20,21].

Educational and awareness gaps regarding the importance and application of genetic information in oncology were also apparent. A shortage of genetic counselors [22,23] and potential hesitancy of physicians to communicate test results, were acknowledged in many regions, factors that may contribute to gaps in screening, testing, and genetically informed care. Similarly, there is a pressing need for education of insurance companies, patients, and the public to ensure uptake of potentially life-saving testing. Ensuring uptake is a multi-factorial challenge that will require engagement of academic institutions, the healthcare industry, patient advocacy groups, plus governmental and non-governmental organizations. Future efforts will need to design a comprehensive action approach that spans four interconnected service pillars: capacity building, affordability, accessibility, and sustainability.

There was a unanimous call for more international collaboration – we envision that transnational medical research can be a bridge to peaceful cooperation that will benefit all populations, albeit political and governmental issues, as well as global conflicts pose challenges. Genomic data are becoming recognized as a valued commodity by many countries, as well as commercial enterprises, and data sharing and exchange are in some cases viewed as a geopolitical national security threat. We note that similar global problems in information exchange have been solved. For example, the Universal Postal Union established in 1874 unified disparate postal services and regulations, allowing international mail to be exchanged freely. Similarly, the internet has become a means of international communication and information exchange. More recently, open-access, de-identified clinical trial and genomic databases have been used by diverse international communities to effectively advance collective knowledge on cancer, which has accelerated therapeutic breakthroughs [24,25,26,27]. Similar strategies to permit free exchange of de-identified genetic data/material and/or potential global harmonization and centralized analysis and interpretation of sequencing data would accelerate discovery and innovation to improve cancer care, while protecting patient privacy and enhancing national interests (e.g., UK Biobank) [28].

Advocacy is critical in continued efforts to ensure equitable and inclusive genetic testing and care. The unified voice of experts, patients, and families will be necessary to overcome the uncertainty of governing bodies to support testing due to cost, lack of understanding, competing priorities, and security concerns.

In summary, this global working group represents a committed effort to engage medical diplomacy to advance genomics for public health and cancer control. From our initial meeting, the group concluded that there is knowledge to be gained from *every* country, as well as opportunities to ensure the best possible care for at-risk populations worldwide, and to advance international shared interest, while fostering transnational collegiality, unity, and engagement. This while this meeting was an important first step in acknowledging the multitude of practical challenges in this area, we recognize there are key ethical and legal issues. While a few members of the working group sit on Ethics Committees of their institutions, we will incorporate experts in international law and bioethics in future discussions. In the long-term, the global barriers to incorporating germline genomics into optimal cancer care identified by this group will inform ongoing research, collaboration, and advocacy for equitable, cost-effective, culturally sensitive, and resource-agnostic genomic medicine for all populations. A follow-up, in-person meeting open to all stakeholders focused on defining specific actions to advance genomics in public health is tentatively scheduled for July 2023.

## Additional File

The additional file for this article can be found as follows:

10.5334/aogh.3967.s1 Supplemental Table 1. Agenda of the Global Oncology Medical Diplomacy Working Group conference of January 5 and 6, 2022.
