# Supplementary material for: Global Oncology Medical Diplomacy Working Group Inaugural Meeting: Defining Worldwide Barriers to Germline Genomics in Cancer Prevention and Management
Source: Ann Glob Health. 2023 Feb 21;89(1):16. doi: 10.5334/aogh.3967 (PMC9951627; doi:10.5334/aogh.3967)
Supplement: Supplemental Table 1. — Agenda of the Global Oncology Medical Diplomacy Working Group conference of January 5 and 6, 2022. [file agh-89-1-3967-s1.pdf]

Supplemental Table 1. Agenda of the Global Oncology Medical Diplomacy Working Group conference of January 5 and 6, 2022

**Wednesday January 5, 2022**		
EST Time	Session	Speakers/Moderarors/Discussants
730 - 731 am	Opening with disclosures	
731 - 734 am	Welcoming Celebrating Humanity Around the Global Video	
734 - 736 am	MSK Leadership welcoming speech	Prof. Lisa DeAngelis
736 - 741 am	UAE Leadership welcoming speech	HE Prof. Amin AlAmiri Asst. Undersecretary at Ministry of Health - UAE
741 - 746 am	Chair opening remarks	Prof. Ghassan Abou-Alfa
746 - 751 am	Co-Chair oprning remarks	Prof. Larry Norton
751 - 836 am	Introduction of Participants	All participants
836 - 856am	Pan-Cancer Germline Assessement	Prof. Zsofia Stadler
856 - 916 am	Questions and Answers	All, led by Prof. Zsofia Stadler
916 - 919 am	Welcoming Celebrating Humanity Around the Global Video	
Concurrent break sessions in separate virtual rooms		
919 - 937 am	Break and interaction time in virtual room 1	Prof. Ghassan Abou-Alfa
	Break and interaction time in virtual room 2	Prof. Larry Norton
937 -1007 am	BRCA Cancer Impact	Prof. David Kelsen
1007 -1027 am	Questions and Answers	All, led by Prof. David Kelsen
1027 -1057 am	Genetic/Familial High-Risk Assessment	Prof. Kenneth Offit
1057 -1117 am	Questions and Answers	All, led by Prof. Kenneth Offit
1117 -1127 am	Day 1 Closing Summary	Prof. Eileen O'Reilly
1127 -1128 am	Day 1 Closing Remarks	Prof. Larry Norton
1128 -1131 am	Welcoming Celebrating Humanity Around the Global Video	

**Thursday January 6, 2022**		
EST Time	Session	Speakers/Moderarors/Discussants
730 - 733 am	Welcoming Celebrating Humanity Around the Global Video	
733 - 740 am	Summary of Day 1	Prof. Larry Norton
740 - 745 am	Introduction to Day 2	Prof. Ghassan Abou-Alfa
Concurrent regional sessions in separate virtual rooms		
745 - 845 am	Africa	
	Co-led by:	Prof. Tanya Trippett
		Prof. Farid Cherbal
	Discussants:	
		Prof. Nermine Kamal
		Prof. Endale Hadgu
		Prof. Maritha Kotze
		Prof. Sheila Mabote
		Prof. Mohsen Mokhtar
		Prof. Mohammed Oukkal
		Prof. Funmi Olopade
		Prof. Lewis Roberts
745 - 845 am	Americas	
	Co-led by:	Prof. Mark Robson
		Prof. Maria Isabel Achatz
	Discussants:	
		Prof. Rosa Alvarez
		Prof. Judy Garber
		Prof. Bruno Nervi

		Prof. Erica Ruiz
		Prof. Andy Seidman
		Prof. Angela Solano
745 - 845 am	Asia	
	Co-led by:	Prof. Bob Li
		Prof. Rebecca Dent
	Discussants:	
		Prof. Samir Fasih
		Prof. Jeong Eun Kim
		Prof. Mrinal Gounder
		Prof. A F M Kamal Uddin
		Prof. Takeshi Kuwata
		Prof. Tony Mok
		Prof. Muhammad Usman Rashid
		Prof. Rajiv Sarin
		Prof. Neelam Siddiqui
		Prof. Bhawna Sirohi
		Prof. Soohwang Teo
		Prof. Joanne Ngeow Yuen Yie
		Prof. Qing Zhou
745 - 845 am	Eurasia	
	Co-led by:	Prof. Rachel Grisham
		Prof. Fabrice Andre
	Discussants:	
		Prof. Gokmen Aktas
		Prof. David Cameron
		Prof. Dilshod Egamberdiev
		Prof. Dilyara Kaidarova
		Prof. Sergei Krasnuy
		Prof. Vsevolod Matveev
		Prof. Sophia Michaelson
		Prof. Eileen O’Reilly
		Prof. Lama Sharara
		Prof. Zsofia Stadler
		Prof. Mehmet Ali Yavuz
745 - 845 am	Middle East	
	Co-led by:	Prof. Larry Norton
		Prof. Shaheenah Dawood
	Discussants:	
		Prof. Hikmat Abdel-Razeq
		Prof. Omalkhair Alkhair
		Prof. Mohammed Algarni
		Prof. Abeer Alsayegh
		Prof. Sultan Al-Sedairy
		Prof. Sana Al-Sukhn
		Prof. Reem Al Sulaiman
		Prof. Basim Ayesh
		Prof. Naji El-Saghir
		Prof. Marwan Ghosn
		Prof. Talia Golan
		Prof. Ephrat Levy-Lahad
		Prof. Moien Kannan
		Prof. David Kelsen

		Prof. Maha Manachi
		Prof. Rami Musallam
		Prof. Philip Philip
		Prof. Maher Saifo
845 - 857 am	Break	
857 - 900 am	Welcoming Celebrating Humanity Around the Global Video	
900 - 920 am	Debriefing from Africa Group	Prof. Tanya Trippett and Prof. Farid Cherbal
920 - 940 am	Debriefing from The Americas Group	Prof. Mark Robson and Prof. Maria Isabel Achatz
940 -1000 am	Debriefing from Asia Group	Prof. Bob Li and Prof. Rebecca Dent
1000 -1020 am	Debriefing from the Eurasia Group	Prof. Rachel Grisham and Prof. Fabrice Andre
1020 -1040 am	Debriefing from the Middle East Group	Prof. Larry Norton and Prof. Shaheenah Dawood
1040 - 1043 am	Welcoming Celebrating Humanity Around the Global Video	
1043 - 1050 am	Break	
1050 -1150 am	Open Discussion and Next Steps	All, led by Prof. Ghassan Abou-Alfa and Prof. Larry Norton
1150 -1155 am	Closing remarks	Prof. Abdulkareem Alolama
1155 -1200 pm	Closing remarks	Prof. Ghassan Abou-Alfa
1200 -1202 pm	Thank you Closing Celebration	
